# *In vitro* activity of fluralaner and commonly used acaricides against *Dermanyssus gallinae* isolates from Europe and Brazil

**DOI:** 10.1186/s13071-018-2956-8

**Published:** 2018-06-25

**Authors:** Emmanuel Thomas, Hartmut Zoller, Gabriele Liebisch, Luis Francisco Angeli Alves, Luis Vettorato, Rafael M. Chiummo, Annie Sigognault-Flochlay

**Affiliations:** 1 MSD Animal Health Innovation GmbH, Zur Propstei, 55270 Schwabenheim, Germany; 2 ZeckLab, Up’n Kampe 3, 30938 Burgwedel, Germany; 3 State University of Western Paraná (UNIOESTE), Cascavel, PR Brazil; 4 MSD Animal Health, São Paulo, Brazil; 5 Merck Animal Health, 2 Giralda Farms, Madison, NJ 07940 USA

**Keywords:** Contact toxicity, Cypermethrin, Deltamethrin, *Dermanyssus gallinae*, Feeding, Fluralaner, Immersion, Phoxim, Propoxur, Spinosad

## Abstract

**Background:**

The poultry red mite *Dermanyssus gallinae* negatively impacts bird welfare and health, and interferes with egg production and quality, while emerging acaricide resistance limits control options. Fluralaner, a novel miticide for administration in drinking water, is approved for control of *D. gallinae* infestations. Mite sensitivity testing is relevant to gauge field isolate susceptibility to available treatments.

**Methods:**

Thirteen *D. gallinae* isolates collected during 2014 through 2016 from farms in Germany, France, Spain and Brazil, and a 2001 laboratory-maintained isolate were used for acaricide contact sensitivity testing. Tested compounds were cypermethrin, deltamethrin, phoxim, propoxur, and the recently available acaricides, spinosad and fluralaner. In each study, at least one isolate was exposed to increasing concentrations of at least one acaricide. In one study, additional testing determined the sensitivity of the 2001 isolate to fluralaner using a mite-feeding test, and of fluralaner, phoxim and spinosad using an immersion test. At least two replicates were used for each dilution. Vehicle and untreated controls were also included.

**Results:**

Based on 90% mortality (LC_90_) values, the laboratory isolate was susceptible to fluralaner (15.6–62.5 parts per million, ppm), phoxim (< 500 ppm), propoxur (< 125 ppm), and deltamethrin (500–1000 ppm). All field isolates remained sensitive to fluralaner concentrations ≤ 125 ppm. Spinosad LC_90_ values for laboratory and field isolates ranged between 2000–4000 ppm. For phoxim, relative to the laboratory isolate, there was reduced sensitivity of two German isolates (LC_90_ up to 4000 ppm) and two French isolates (> 4000 ppm). An isolate from Spain demonstrated reduced sensitivity to phoxim, propoxur and deltamethrin; an isolate from Brazil showed reduced sensitivity to propoxur and cypermethrin. Mite LC_90_ when exposed to fluralaner by blood feeding was < 0.1 ppm.

**Conclusions:**

Contact sensitivity testing indicated apparent resistance to at least one of phoxim, deltamethrin, cypermethrin and propoxur in 13 field isolates from Europe and Brazil. All isolates were highly susceptible to fluralaner. Fluralaner was approximately 1000 times more active by feeding than by contact. Fluralaner’s distinct mode of action and efficacy against isolates largely refractory to those acaricides, makes it a promising option for the control of *D. gallinae* infestations of poultry*.*

## Background

The poultry red mite *Dermanyssus gallinae* (De Geer, 1778) is an important ectoparasite of poultry that negatively impacts poultry health and welfare, reduces egg production and egg quality, and is a potential pathogen vector [1–8]. Additionally, mite infestations of poultry workers and of city dwellers with no connections to poultry have been reported as a significant public health concern [9–11]. An ubiquitous hematophagous ectoparasite, *D. gallinae* seeks its hosts at night, taking blood meals that typically last for less than two hours, after which mites retreat into cracks and crevices of nest boxes or other suitable sites within the wider surroundings of a poultry housing unit [6, 7, 12–14]. At temperatures of 10–37 °C (with 25 °C considered optimum), and relative humidity ranging from 70 to 90%, the life-cycle of *D. gallinae* may be as short as 7 days [8, 15–17]. Such conditions may result in a weekly doubling of the mite population in poultry houses, with up to 500,000 mites potentially infesting one hen in severe cases [5, 7, 14].

Until recently, the control of the poultry red mite has been largely dependent upon the use of sprayed acaricides, such as organophosphates, carbamates, macrocyclic lactones, pyrethroids, and spinosyns [8, 14]. However, increasingly restrictive legislation has presented a practical constraint for producers attempting to control this parasite, in some cases leading to illegal or off-label use of banned pesticides, such as carbaryl and fipronil, with risk of food-chain residues [2, 4, 18, 19]. Moreover, repeated acaricide applications, particularly if at incorrect dosages, has led to the emergence of resistance in poultry red mite populations [4, 8]. Resistance to approved and non-approved acaricides, including alpha-cypermethrin, bifenthrin, carbamates, carbaryl, cypermethrin, deltamethrin, dichlorodiphenylytrichloroethane (DDT), dichlorvos, fenitrothion, fipronil, flumethrin, flurathiocarb, malathion, permethrin, phenothrin, tetramethrin and trichlorfon, has been reported from many countries, including Czechoslovakia, France, Italy, Korea, Montenegro, Poland, Serbia and Sweden [3, 18, 20–24]. Along with a move to housing systems which favour mite proliferation, an outcome of the European Union (EU) directive banning the use of traditional cages since 2012, the emergence of resistance complicates parasite control measures, potentially leading to higher treatment frequencies and increased risk of egg residues [4, 16]. As a result the control of *D. gallinae* by effective and safe treatments has been a largely unmet need for nearly two decades [4].

During 2017, the isoxazoline compound fluralaner was approved for the control of *D. gallinae* on EU poultry operations [25]. This approval was the outcome of a development programme in which fluralaner, administered in drinking water at a dose rate of 0.5 mg/kg on two occasions with a 7-day interval, was shown to be safe and to have a zero day egg withdrawal period following treatment. Mite killing has been shown to begin within four hours following an initial fluralaner administration, and on commercial farms in France, Germany and Spain efficacy reached 97.8–100% as soon as two days following the second administration [26, 27]. Fluralaner selectively targets distinct binding sites on insect and arachnid ligand-gated chloride channels in the nervous system, leading to parasite paralysis and death. This mode of action is different from that of any other poultry acaricide, and cross-resistance between fluralaner and traditional chemicals which act by contact is unlikely [28].

To determine the sensitivity of field isolates of *D. gallinae* to fluralaner, and to assess the sensitivity of these isolates to commonly used mite control acaricides, six *in vitro* laboratory studies were initiated. Mite isolates were collected for testing from commercial poultry operations in Europe and Brazil. Comparator active ingredients were spinosad, phoxim, propoxur, and the pyrethroids cypermethrin and deltamethrin.

## Methods

### Study design

**Fig. 1:**
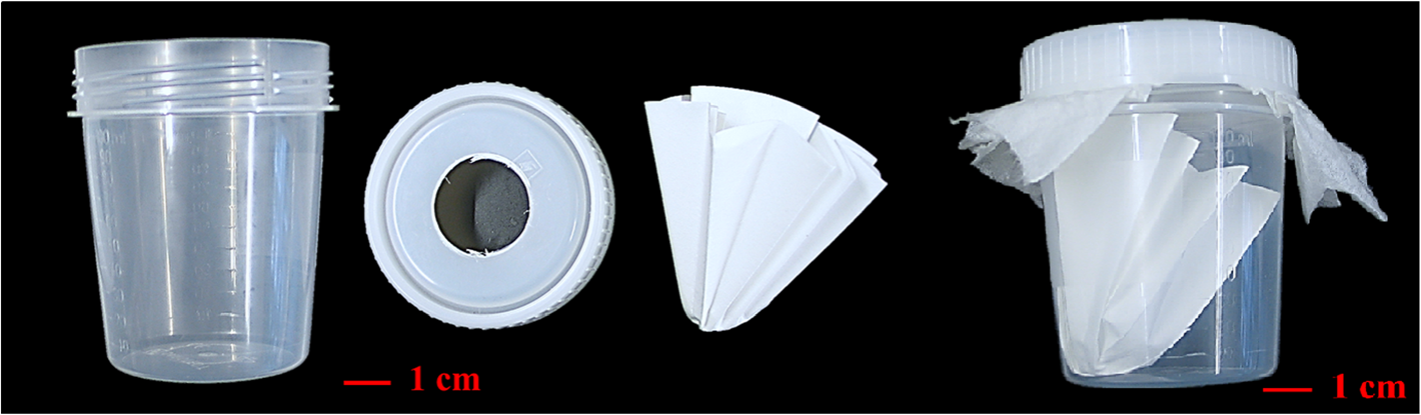
Plastic container used for collection of mites from poultry houses

Across six separate studies, 12 field isolates were freshly collected between May 2014 and July 2015 from commercial layer, breeder, and pullet farms located in Germany, France and Spain, and in September 2016 one isolate was collected from a layer farm in Brazil. No chemical acaricidal treatment had been used in the sampled houses for at least two months prior to mite collection. In Studies 1 through 5, mites were brushed from typical refuge areas into plastic containers that contained filter paper to absorb humidity, and that had a lid-hole covered with air-permeable paper to preclude mite escape (Fig. 1). In Study 6, mites were collected in traps (Avivet, Lunteren, Netherlands) that had been placed in laying houses for 48 hours prior to collection. Following mite collection containers were taken directly or shipped overnight to the testing laboratory. The laboratory isolate used in Studies 1 and 5 was originally collected from a German commercial layer farm in 2001, and had been maintained at MSD Animal Health Innovation (Schwabenheim, Germany).

Fluralaner was sourced from MSD Animal Health Innovation; commercially available phoxim, deltamethrin, and propoxur were sourced from Sigma-Aldrich Chemicals (Allentown, USA); spinosad from APIN Chemicals (Abingdon, UK), and cypermethrin from Tagros Chemicals (Chennai, India). All compounds tested were of technical grade greater than 95%.

The *in vitro* contact sensitivity of each of the 13 *D. gallinae* field isolates and a laboratory isolate was tested in at least one of three separate laboratories: MSD Animal Health Innovation GmbH, Schwabenheim, Germany (Study 1; field isolate 1); ZeckLab, Burgwedel, Germany (Studies 2 to 5; field isolates 1–12), and the Agricultural Biotechnology Laboratory, State University of Western Paraná, Cascavel, PR, Brazil (Study 6, field isolate 13) (Table 1). Study 1 also included a feeding test and an immersion contact test. In all studies, after removal from test media and incubation, mite mortality (MM) was assessed using a binocular microscope to count all mites, living and dead. If present, moribund mites were counted as dead. Nymphal and adult stages were not differentiated.

**Table 1:** Study design summary

Study	Assay	Concentrations tested (ppm)
1	Contact	Fluralaner, phoxim, spinosad: 3.9–4000
	Immersion	Phoxim, spinosad: 2.0–4000; fluralaner: 0.3–4000
	Feeding	Fluralaner 10^-7^–10
2	Contact	Deltamethrin^a^: 250, 125, 62.5
3, 4	Contact	Fluralaner: 3.9–62.5; spinosad: 250– 4000; phoxim: 125–2,000; propoxur, deltamethrin: 62.5–1000
5	Contact	Fluralaner: 15.6–125; spinosad, phoxim: 500–4000; propoxur, deltamethrin: 125–1000
6	Contact	Fluralaner, propoxur, cypermethrin: 1.95–1000

^a^Due to the low number of live mites available, only deltamethrin was tested

### Sensitivity testing

#### Contact sensitivity

Contact sensitivity assays using impregnated filter paper were modifications of the Larval Package Test recommended by the Food and Agriculture Organization (FAO) for resistance testing of tick larvae [29]. Two replicate sets of filter paper were assessed for each control and each concentration of active. In Studies 1 and 6, actives were dissolved in 2.5 ml dimethyl sulfoxide to obtain a stock solution, 2 ml of which were mixed with 38 ml of deionized water to obtain a test concentration of 1000 ppm. Five ml of test solution or solvent control were pipetted onto a filter paper (185 mm diameter) and dried at room temperature for approximately 24 h. From 60 to 400 mites in Study 1, and approximately 50 mites in Study 6, were placed onto each filter paper which was then triple-folded and sealed with a tacker, allowing direct contact between mites and the test solutions. After incubation for approximately 48 h, in Study 1 at approximately 22 °C and 90% relative humidity (RH), and in Study 6 at 23 ± 1 °C and 80 ± 5% RH, the filter papers were opened for assessment of the mites.

**Fig. 2:**
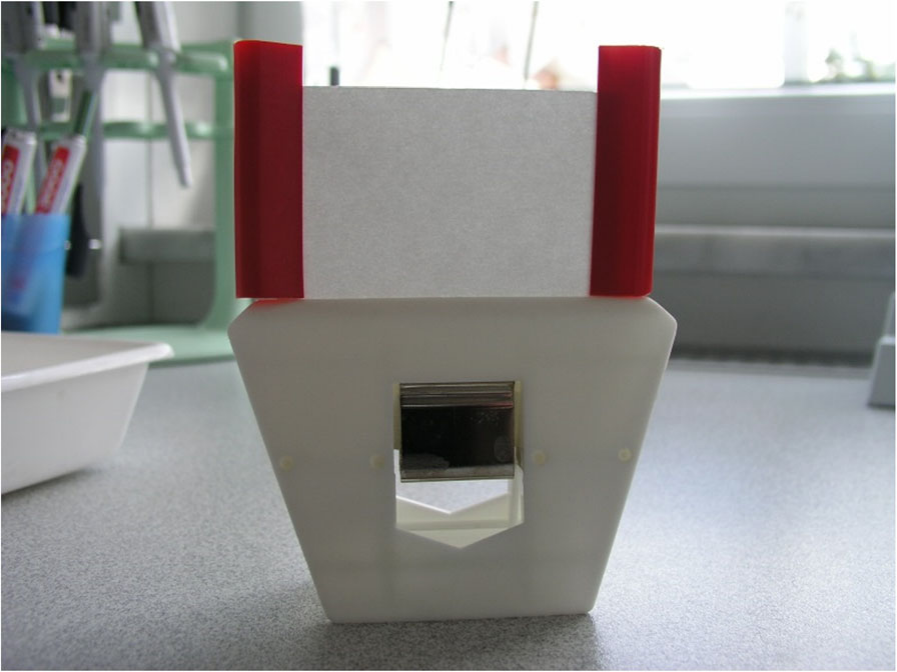
Filter paper package used in the contact tests

In Studies 2 to 5, actives were dissolved in 1-butanol + olive oil (DAB10) to obtain a 1% stock solution (100 mg substance/10 ml solvent). Test concentrations were prepared by further 1:2 dilutions with deionized water. Filter papers (8 × 10 cm) were coated with 800 μl of the final test solutions or with solvent only (control), and left for drying for 48 h at room temperature. Impregnated papers were folded and closed with clamps on two sides. Approximately 100 mites were placed into the opening of each filter paper which was then sealed and placed in a holding apparatus (Fig. 2) for 48 h at 40 to 50% RH and temperature of 20 to 23 °C. Diluent with no miticide was used as a negative control, and an additional control was not exposed to either vehicle or active.

#### Immersion contact and feeding tests (Study 1)

For the immersion (larval sandwich) test, mites were placed between two filter papers and 5 ml of test solution was added. Mites were immersed (between the filter papers) in fluralaner (0.031 to 4000 ppm), phoxim or spinosad solutions (each 2 to 4000 ppm). After approximately three minutes, the solution was poured off, the filter papers were dried with towelling paper and the mites were then placed onto a fresh triple folded filter paper that was sealed (as a sachet). After incubation for 48 h, at approximately 22°C and 90% RH, all mites were counted. There were two replicates for each test compound and the two controls, one containing DMSO solvent and one untreated.

For the feeding test, between 100 and 150 mites were transferred into plastic vials (diameter 20 mm), each closed with a membrane (Parafilm®) that had been treated with mite-attractant solution prepared from an ethanol-butyric acid extraction of a mixture containing minced chicken skin and feathers. The vials were then placed into a glass dish containing 2 ml of a warmed blood solution comprised of a mixture of defibrinized sheep blood and stock solution, and fluralaner at concentrations ranging from 10^-7^ to 10^1^ ppm. Control mites were exposed to either DMSO solution or to blood only. After two hours feeding in darkness, engorged mites were collected, transferred into a glass tube containing a paper strip, closed with an air-permeable lid, and incubated for 24 h at approximately 22 °C and 90% RH. The test included two controls (one DMSO solvent and one untreated control), and was carried out in three replicates. Phoxim was not included in the feeding test because of its volatility.

### Assessments

The MM, expressed as a percentage and calculated for each test concentration approximately 48 h after contact exposure (studies 1 to 6), or 24 h after feeding (study 1 only), was calculated as follows:

Mite mortality = (n_dead_/n_counted_) × 100

where n_dead_ is the arithmetic mean (of all replicas) of dead mites per test concentration; n_counted_ is the arithmetic mean (of all replicas) of counted mites per test concentration.

The per cent MM was calculated using the formula:

$$ Efficacy\kern0.28em \left(\%\right)=\frac{M{M}_T-M{M}_C}{100-{MM}_C}\times 100 $$

where MM_C_ is the per cent MM of mites exposed to the control solution and MM_T_ is the mortality of mites exposed to the test concentration. For Studies 1 and 6, a mean of the solvent and the untreated control was used as MM_C_.

In Study 1 the 90% lethal concentrations (LC_90_) were calculated using probit analysis (SAS® release 9.2). In Studies 2 to 5 the low number of tested concentrations limited LC_90_ estimations to a certain range_._ Results therefore focus on concentrations where mortalities exceeded 90%, with comparisons to LC_90_ values calculated in Study 1.

## Results

**Fig. 3:**
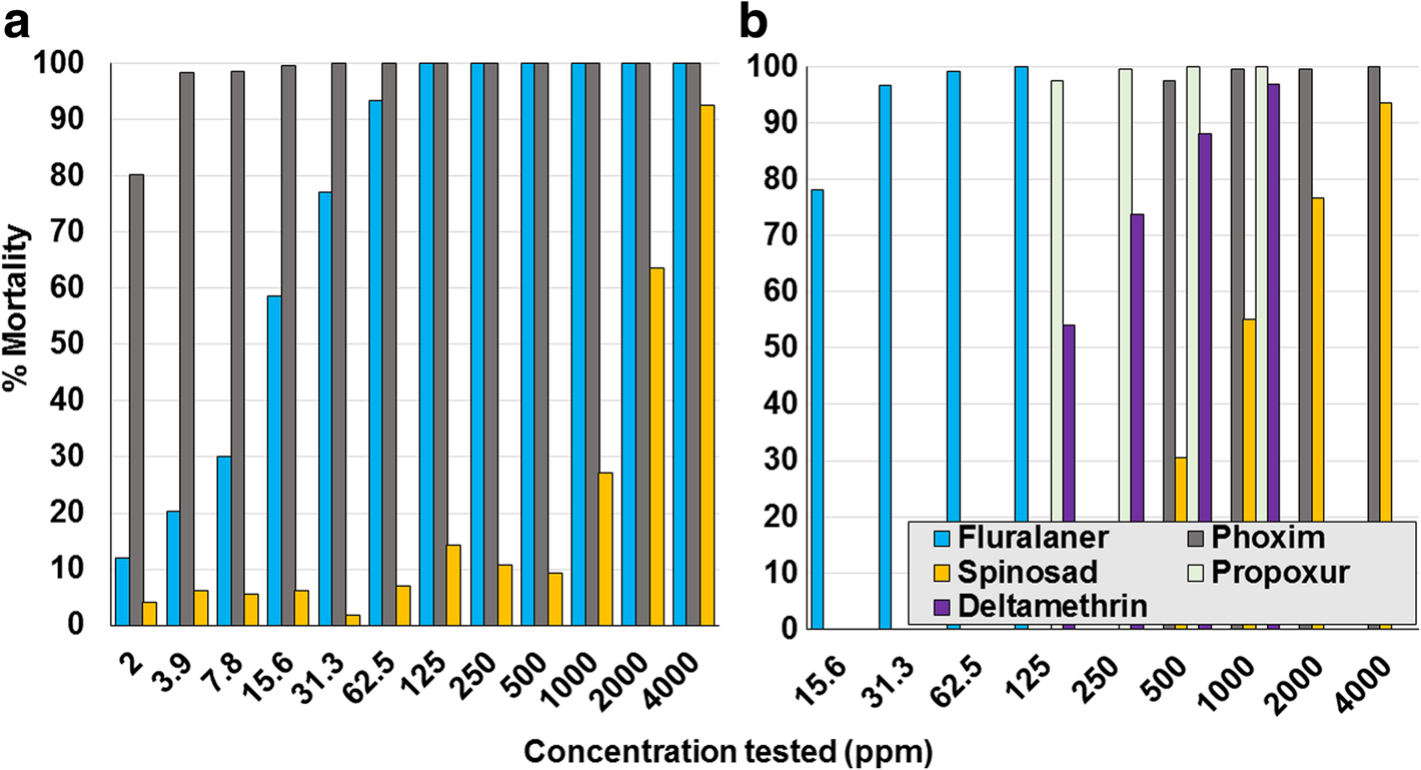
Assay results from the two laboratories that tested contact sensitivity of the 2001 laboratory isolate. **a** Laboratory 1. **b** Laboratory 2

**Fig. 4:**
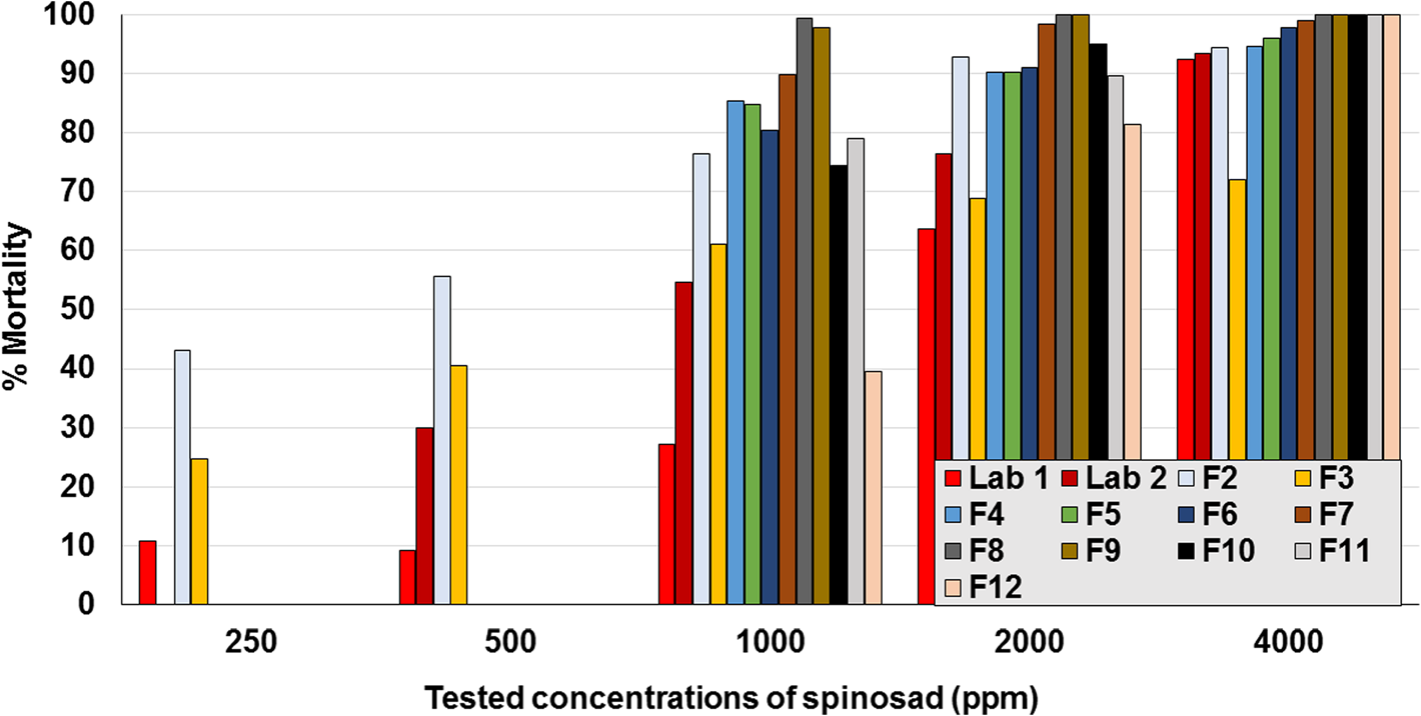
Per cent mortality of *Dermanyssus gallinarum* following contact exposure to concentrations of spinosad (Lab 1 and Lab 2 denote the results of separate laboratories for the field strain isolated in 2001; F denotes field isolate)

**Fig. 5:**
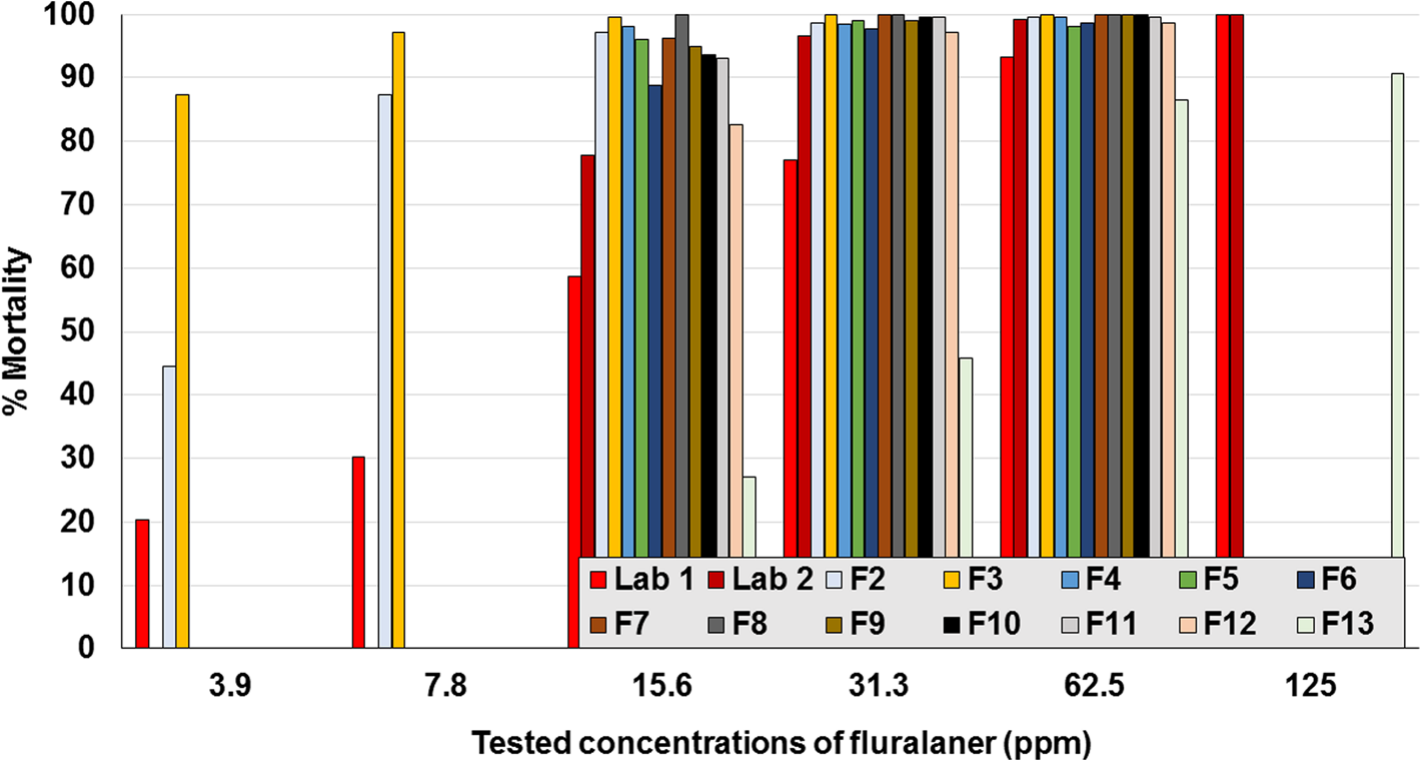
Per cent mortality of *Dermanyssus gallinarum* following contact exposure to concentrations of fluralaner (Lab 1 and Lab 2 denote the results of separate laboratories for the field strain isolated in 2001; F denotes field isolate)

The MM in controls ranged between 0.5–16.1% across contact assay studies. Overall numbers of moribund mites were too few to make any notable difference to the results, and so only the counts derived from dead mites are presented. Based on LC_90_ values, the laboratory isolate was susceptible to fluralaner (range 15.6–62.5 ppm), spinosad (2000–4000 ppm), phoxim (2.6 and < 500 ppm), propoxur (< 125 ppm), and deltamethrin (500–1000 ppm) (Fig. 3a, b; Tables 2, 3, 4, 5 and 6). The two laboratories that assayed the German isolate from 2001 (laboratory isolate) returned similar contact sensitivities to fluralaner, spinosad and phoxim, indicating a consistency of results despite some differences in the testing methodologies. Field isolates were sensitive to spinosad concentrations of 2000–4000 ppm (Fig. 4). Laboratory and field isolates showed similar sensitivity to fluralaner (≤ 125 ppm) (Fig. 5).

**Table 2:** Lethal concentration, based on 90% mortality (LC_90_), of tested actives against different *D. gallinae* isolates

Study	Isolate (year, country)	LC_90_ (ppm)				
		Fluralaner	Pyrethroids	Phoxim	Propoxur	Spinosad
1	Lab 1 (2001, Germany)	62.5	nt	2.0–3.9	nt	2000–4000
2	Field 1 (2014, Germany)	nt	> 250 (D)	nt	nt	nt
3	Field 2 (2014, France)	7.8–15.6	> 1000 (D)	> 1000	> 1000	1000–2000
4	Field 3 (2015, Spain)	3.9–7.81	> 1000 (D)	> 2000	> 1000	> 4000
5	Field 4 (2015, Germany)	< 15.6	nt	2000–4000	nt	1000–2000
	Field 5 (2015, France)	< 15.6	nt	> 4000	nt	1000–2000
	Field 6 (2015, France)	15.6–31.3	nt	> 4000	nt	1000–2000
	Field 7 (2015, Germany)	< 15.6	nt	< 1000	nt	1000–2000
	Field 8 (2015, Spain)	< 15.6	nt	2000–4000	nt	< 1000
	Field 9 (2015, France)	< 15.6	nt	2000–4000	nt	< 1000
	Field 10 (2015, Germany)	< 15.6	nt	1000–2000	nt	1000–2000
	Field 11 (2015, Germany)	< 15.6	nt	1000–2000	nt	1000–2000
	Field 12 (2015, Germany)	15.6–31.3	nt	< 1000	nt	2000–4000
	Lab 2 (2001, Germany)	15.6–31.3	500–1000 (D)	< 500	< 125	2000–4000
6	Field 13 (2016, Brazil)	125	> 1000 (C)	nt	> 1000 (C)	nt

*Abbreviations*: Lab 1 and Lab 2, same laboratory isolate tested in different laboratories; nt, not tested; D, deltamethrin; C, cypermethrin

**Table 3:** Per cent *in vitro* mortality of laboratory and field isolates of *Dermanyssus gallinae* exposed to fluralaner

Isolate	Concentration (ppm)									
	2.0	3.9	7.8	15.6	31.3	62.5	125	1000	2000	4000
Lab 1	1.2	20.4	30.2	58.6	77.1	93.3	100	100	100	100
Lab 2			77.9	96.6	99.2	100				
Field 2	44.6	87.3	97.2	98.6	99.6					
Field 3	87.3	97.2	99.6	100	100					
Field 4			98.0	98.5	99.5					
Field 5			96.0	99.0	98.1					
Field 6			88.7	97.7	98.6					
Field 7			96.2	100	100					
Field 8			100	100	100					
Field 9			95.0	99.0	100					
Field 10				93.6	99.5	100				
Field 11				93.1	99.6	99.6				
Field 12				82.6	97.1	98.6				
Field 13				27.1	45.8	86.5	90.6	96.9

**Table 4:** Per cent *in vitro* mortality of laboratory and field isolates of *Dermanyssus gallinae* exposed to spinosad

Isolate	Concentration (ppm)											
	2.0	3.9	7.8	15.6	31.2	62.5	125	250	500	1000	2000	4000
Lab 1	4.1	6.1	5.5	6.2	1.8	7.0	14.2	10.8	9.2	27.2	63.7	92.5
Lab 2									29.9	54.6	76.4	93.5
2								43.2	55.7	76.5	92.9	94.5
3								24.7	40.5	61.0	68.9	72.0.
4										85.4	90.3	94.7
5										84.8	90.3	96.0
6										80.5	91.0	97.8
7										89.8	98.4	99.0
8										99.4	100	100
9										97.7	100	100
10										74.4	95.0	100
11										79.1	89.6	100
12										39.5	81.5	100

Commercial rate for application of spinosad is *c.*2000–4000 ppm

**Table 5:** Per cent *in vitro* mortality of laboratory and field isolates of *Dermanyssus gallinae* exposed to phoxim

Isolate	Concentration (ppm)											
	2.0	3.9	7.8	15.6	31.2	62.5	125	250	500	1000	2000	4000
Lab 1	80.3	98.4	98.6	99.6	100	100	100	100	100	100		
Lab 2									97.5	99.6	99.6	100
Field 2							0.2	3.6	5.5	6.9		
Field 3								14.4	17.3	7.0	46.8	
Field 4										23.7	84.7	91.5
Field 5										21.7	27.3	46.2
Field 6										4.0	15.0	80.0
Field 7										93.1	100	100
Field 8										30.4	56.1	98.5
Field 9										17.6	77.5	100
Field 10										55.8	95.4	98.6
Field 11										48.4	94.0	100
Field 12										98.7	100	100

**Table 6:** Per cent *in vitro* mortality of laboratory and field isolates of *Dermanyssus gallinae* following exposure to either propoxur or pyrethroids (deltamethrin for isolates from France and Spain, cypermethrin for Brazil isolate)

Isolate	Propoxur concentration (ppm)					Pyrethroid concentration (ppm)				
	62.5	125	250	500	1000	62.5	125	250	500	1000
Lab 2		97.5	99.6	100	100		53.5	73.5	88.1	96.9
Field 1						36.9	47.1	52.1	nt	nt
Field 2	7.6	11.6	10.9	13.2	13.8	3.3	6.4	3.1	3.2	4.9
Field 3	7.7	8.1	6.4	5.2	12.4	16.3	11.7	11.0	13.8	12.9
Field 13	17.7	30.2	15.6	15.6	19.8	2.1	3.1	4.1	7.3	15.6

*Notes*: Commercial rate for application of propoxur is 1000 ppm. Commercial rates for application of deltamethrin and cypermethrin are 250 and 150 ppm, respectively. For field isolate 13, for propoxur concentrations < 62.5 ppm, mite mortality ranged from 0.0 to 12.5% at 1.95 to 31.3 ppm, respectively. For cypermethrin mortality ranged from 0.0 to 1.0% at 1.95 to 31.3 ppm, respectively

**Fig. 6:**
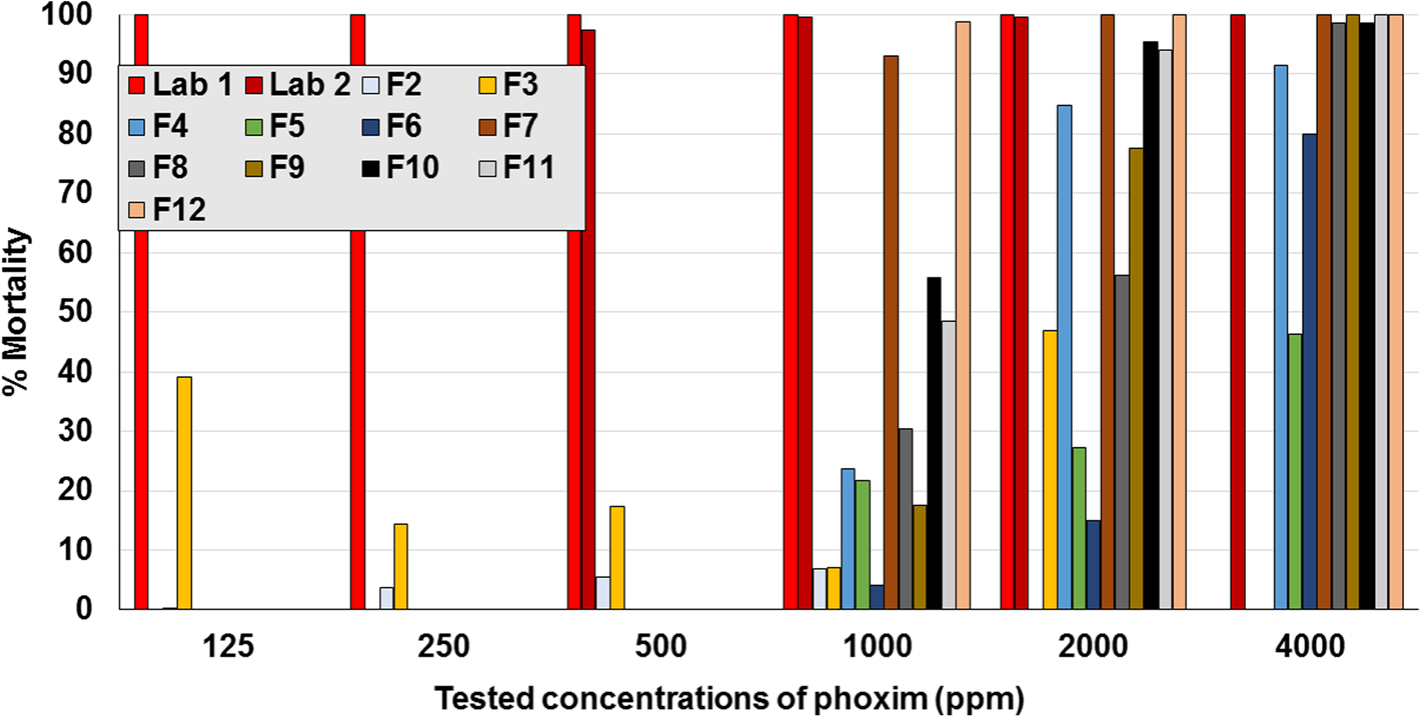
Per cent mortality of *Dermanyssus gallinarum* following contact exposure to concentrations of phoxim (Lab 1 and Lab 2 denote the results of separate laboratories for the field strain isolated in 2001; F denotes field isolate)

For phoxim, relative to the laboratory isolate (LC_90_ 2.6 ppm), there was an apparent decreased contact sensitivity of two German field isolates for which LC_90_ had increased to between 1000–2000 ppm (field isolates 10 and 11), and one isolate for which the LC_90_ lay between 2000–4000 ppm (field isolate 4) (Tables 2 and 5; Fig. 6). The phoxim LC_90_ for French field isolates 5 and 6 were greater than 4000 ppm, a concentration at which mortalities were just 46.2 and 80.0%, respectively. For the French isolate (Field 2), MM at the maximum tested phoxim concentration (1000 ppm) was 6.9%. This isolate also showed reduced sensitivity to both propoxur and deltamethrin. Field isolate 3 from Spain also demonstrated similar reduced sensitivity to phoxim, propoxur and deltamethrin. Reduced sensitivity was demonstrated by field isolate 13 from Brazil to propoxur and cypermethrin: contact exposure to 250 ppm resulted in mortality of 15.6 and 4.1%, respectively, only marginally increasing to 19.8% and 15.6% at 1,000 ppm, the highest tested exposure (Table 6).

**Fig. 7:**
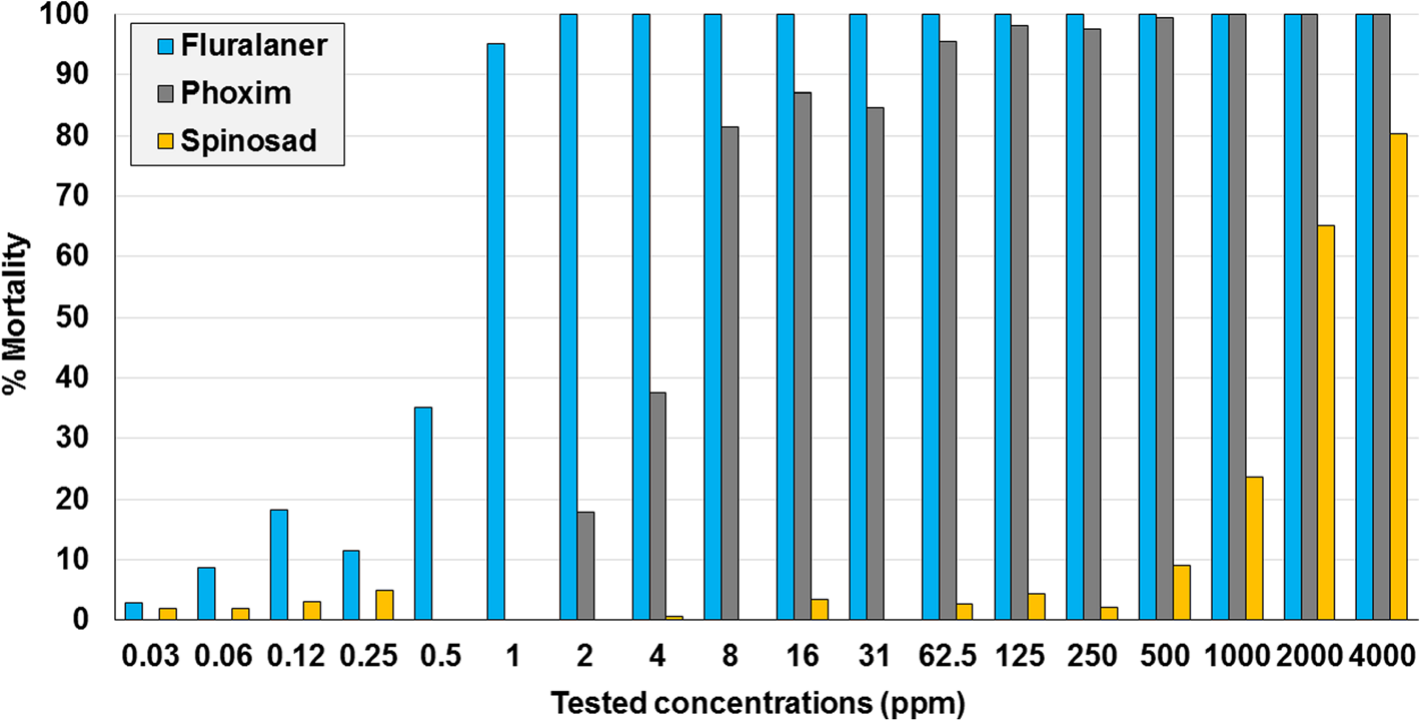
Per cent mortality of the laboratory isolate of *Dermanyssus gallinarum* following 3-minute immersions in concentrations of fluralaner, phoxim and spinosad

The MM following immersion in concentrations of fluralaner and phoxim exceeded 90% at 1 ppm and 62.5 ppm, respectively. The peak measured MM following immersion in increasing concentrations of spinosad was 80.4% at 4000 ppm (Fig. 7). In the feeding test, MM was 100% following exposure to conentrations of 0.1 ppm or greater. Based on the measured value leading to LC_50_ and LC_90_, MM from exposure to fluralaner was greater in the feeding test than in either of the contact tests (filter paper and immersion) (Table 7).

**Table 7:** Calculated 50% (LC_50_) and 90% (LC_90_) values (ppm) of a laboratory strain of *D. gallinae* exposed *in vitro* to fluralaner, phoxim and spinosad

Test	Compound	LC_50_	LC_90_
Filter paper contact	Fluralaner	11.62	49.93
	Phoxim	0.72	2.63
	Spinosad	1524	*c.*4000^a^
Immersion	Fluralaner	0.52	1.57
	Phoxim	4.25	30.44
	Spinosad	2311	> 4000^a^
Feeding	Fluralaner	0.0125	< 0.1^a^

^a^These values could not be estimated using the mathematical model

## Discussion

Multiple reports from individual countries have indicated that *D.gallinae* resistance to many acaricidal classes (organophosphates, pyrethroids, carbamates) is widespread, and is a contributing factor to the increased prevalence of poultry red mites [4, 8, 22, 24, 30]. This is the first report to describe reduced sensitivity to a range of acaricides across different countries. When compared to the commercial product label rates, the LC_90_ values described in this report provide further evidence that poultry red mite resistance to multiple chemical groups has developed under commercial production conditions. From the 11 field isolates originating from layer and breeder farms in France, Spain and Germany, six had a LC_90_ for phoxim exceeding the recommended concentration for commercial use. Deltamethrin and cypermethrin were tested on one Brazilian and three EU field isolates, and all had LC_90_ values exceeding the commercial product label rates of 250 and 100–200 ppm [31, 32]. The same was observed with propoxur for which the LC_90_ of the three isolates from Europe and Brazil exceeded 1000 ppm, a concentration that, in the authors experience, was used in poultry prior to its withdrawal from the European market.

In this report, three methods were used: a filter paper test corresponding to the spray application used for the traditional treatments which are contact acaricides; an immersion test; and a feeding test which is relevant to fluralaner because mites are exposed while feeding on treated birds. Irrespective of the test method, fluralaner was consistently active at low concentrations against all the tested *D*. *gallinae* isolates. The greatest activity of fluralaner was observed in the feeding test at extremely low lethal concentrations (LC_90_ < 0.1 ppm), approximately 1000 times lower than that obtained during the filter paper contact test. The latter test thus provides a “worst case scenario” with reference to fluralaner’s systemic mode of action, especially when compared to results of other acaricides with a contact mode of action. The value of more potent systemic than contact activity for fluralaner is that the innovative mode of administration (in drinking water) enables the use of a lower and more precise amount of active, when compared with a spray application.

The spinosad LC_90_ of up to 4000 ppm for the laboratory and EU field isolates with MM assessed after 48 hours, indicates that this chemical is less potent than the other tested acaricides, including fluralaner, and is consistent with previous sensitivity reports [33]. These concentrations are close to, or even exceed the spinosad recommended dose rate. The killing activity of spinosad has been reported to increase beyond 48 hours after exposure, although even after five days a concentration of 2000 ppm failed to achieve a 90% mortality of adult mites [33].

The present studies assessed MM following exposure of a 2001 isolate, and isolates from 2014 to 2015, to phoxim, propoxur and spinosad. Using the same method at the same laboratory, the results show a declining sensitivity of field isolates to phoxim and propoxur. No change was observed for spinosad which was only launched in 2012. This supports the belief that resistance to older compounds has continued to build during the last decade [8].

Results from the studies described in this report show that fluralaner is active against poultry red mites, isolated from commercial operations, showing decreased susceptibility to the most commonly used acaricides. These findings are consistent with reports of fluralaner efficacy against *Ornithonyssus sylviarum*, the northern fowl mite, particularly prevalent in North and Latin America, at similar exposure rates as those to which *D. gallinae* is sensitive [34]. They also align with previous results showing that fluralaner has been shown to overcome resistance in fleas, ticks or sea lice against phenylpyrazoles, amidines, organophosphates, cyclodienes, macrocyclic lactones, phenylpyrazoles, benzophenyl ureas, pyrethroids and carbamates [35].

## Conclusions

The studies reported in this paper demonstrate that the poultry red mite *D. gallinae* is highly susceptible to the novel poultry acaricide fluralaner, with much greater sensitivity shown by exposure through feeding on blood than by contact or immersion. This differential sensitivity toward exposure through feeding aligns with fluralaner’s oral administration for systemic activity against mites. Similar susceptibility to fluralaner was observed for European and Brazilian field isolates showing apparent resistance to phoxim, deltamethrin, cypermethrin and propoxur. The different mode of action than that of other poultry acaricides, and the efficacy demonstrated against isolates largely refractory to those acaricides, makes fluralaner a promising option for the control of *D. gallinae*, helping to reduce or avoid the costly effects of this damaging parasite.

## Abbreviations

DDT: Dichlorodiphenylytrichloroethane
FAO: Food and Agriculture Organisation
LC_90_: Lethal concentration that causes 90% mortality
MM: Mite mortality
Ppm: Parts per million
RH: Relative humidity
